# KIR2DS2+ NK cells in cancer patients demonstrate high activation in response to tumour-targeting antibodies

**DOI:** 10.3389/fonc.2024.1404051

**Published:** 2024-09-02

**Authors:** Lara V. Graham, Jack G. Fisher, Amber D. P. Doyle, Ben Sale, Luis Del Rio, Albert J. E. French, Neema P. Mayor, Thomas R. Turner, Steven G. E. Marsh, Mark S. Cragg, Francesco Forconi, Salim I. Khakoo, Matthew D. Blunt

**Affiliations:** ^1^ School of Clinical and Experimental Sciences, University of Southampton, Southampton, United Kingdom; ^2^ School of Cancer Sciences, University of Southampton, Southampton, United Kingdom; ^3^ Anthony Nolan Research Institute, Royal Free Hospital, London, United Kingdom; ^4^ Department of Academic Haematology, University College London (UCL) Cancer Institute, London, United Kingdom; ^5^ Antibody and Vaccine Group, Centre for Cancer Immunology, Faculty of Medicine, University of Southampton, Southampton, United Kingdom; ^6^ Haematology Department, Cancer Care Directorate, University Hospital Southampton National Health Service (NHS) Trust, Southampton, United Kingdom

**Keywords:** KIR2DS2, NK cells, cancer, immunotherapy, KIR, ADCC

## Abstract

Strategies to mobilise natural killer (NK) cells against cancer include tumour-targeting antibodies, NK cell engagers (NKCEs) and the adoptive transfer of *ex vivo* expanded healthy donor-derived NK cells. Genetic and functional studies have revealed that expression of the activating killer immunoglobulin-like receptor KIR2DS2 is associated with enhanced function in NK cells from healthy donors and improved outcome in several different malignancies. The optimal strategy to leverage KIR2DS2+ NK cells therapeutically is however currently unclear. In this study, we therefore evaluated the response of KIR2DS2-expressing NK cells to activation against cancer with clinically relevant tumour-targeting antibodies and following *ex vivo* expansion. We identified that KIR2DS2^high^ NK cells from patients with chronic lymphocytic leukaemia and hepatocellular carcinoma had enhanced activation in response to tumour-targeting antibodies compared to KIR2DS2- NK cells. However, the superior function of healthy donor derived KIR2DS2^high^ NK cells was lost following *ex vivo* expansion which is required for adoptive transfer-based therapeutic strategies. These data provide evidence that targeting KIR2DS2 directly in cancer patients may allow for the utilisation of their enhanced effector function, however such activity may be lost following their *ex vivo* expansion.

## 1 Introduction

Natural killer (NK) cells are cytotoxic innate lymphocytes with an increasingly recognised role in the control of cancer via direct lysis of target cells and promotion of the anti-cancer immune response through release of proinflammatory cytokines such as IFNγ (1). Due to their expression of the Fc gamma receptor (FcγR) CD16A, NK cells can also contribute to the anti-tumour functions of certain monoclonal antibodies (mAbs) via antibody dependent cellular cytotoxicity (ADCC) (2). NK cells represent powerful cellular therapeutic tools for controlling tumour growth however, in cancer patients, NK cells can become exhausted and/or dysfunctional (3–5). The adoptive transfer of NK cells from healthy donors with or without the expression of a chimeric antigen receptor (CAR) (6, 7) can overcome this issue and CAR-NK cells are currently demonstrating an improved safety profile compared to CAR-T cells in clinical trials, with no graft versus host disease (GvHD), cytokine release syndrome or neurotoxicity reported to date (8, 9). However, NK cells sourced from healthy donors are heterogeneous and the expression of activating and inhibitory cell surface receptors and potency of anti-tumour responses is variable, presenting a limitation to the implementation of effective allogeneic NK adoptive transfer therapies in the clinic (6, 7, 10). To overcome this issue, the identification of genetic and phenotypic NK cell traits associated with superior function could inform optimal donor selection and/or parameters to monitor during *ex vivo* expansion. Indeed, the selective expansion of NK cells from donors with high expression of the activating receptor NKG2C has recently been shown to improve anti-tumour activity *in vivo*, and associate with enhanced cytotoxicity against primary leukemic blast cells *ex vivo* (11). This exemplifies the potential utilisation of NK cell subpopulations expressing specific receptors to aid donor selection and expansion strategies.

A critical receptor family for controlling NK cell activation is the Killer-cell Immunoglobulin-like Receptor (KIR) family which is made up of activating and inhibitory isoforms (12, 13). Ligation of inhibitory KIR with self-HLA prevents the killing of healthy host cells by NK cells (14) and HLA downregulation by malignantly transformed cells can unleash NK cell cytotoxicity, a paradigm known as missing-self (15). Immunogenetic studies have revealed that the activating receptor KIR2DS2 is associated with improved outcomes across multiple cancer types (16–20) and improved outcome following cord blood (21) or peripheral blood stem cell (22) transplant for patients with haematological malignancies. However, the high sequence homology between the activating and inhibitory KIR has hindered investigations into the relationship between KIR2DS2 and survival in cancer patient RNA-sequencing datasets. KIR2DS2 is known to bind HLA-C in combination with virus-associated peptides (13), although no cancer specific ligands have been reported to date. Furthermore, this is supported by *in vitro* studies which have demonstrated that KIR2DS2 is associated with enhanced effector functions (21, 23–25) and a transcriptional profile enriched for NK cell cytotoxicity-associated genes (25). It is currently unclear however whether the enhanced functional capacity of KIR2DS2+ NK cells is retained in patients with cancer or following their *ex vivo* expansion. This information is critical to allow for the effective utilisation of KIR2DS2+ NK cells in cancer and in this study we aimed to address this.

## 2 Methods

### 2.1 Primary patient samples and cell lines

Healthy donor (male and female, age range 22-60 years) peripheral blood mononuclear cells (PBMCs) were obtained with full ethical approval from the National Research Ethics Committee (reference 06/Q1701/120). HCC patients (**Supplementary Table 1**) were recruited from the outpatient clinic at Southampton General Hospital and provided informed consent and approved by NRES Committee South Central-Southampton-B 12/SC/0521. CLL samples (**Supplementary Table 2**) were obtained from patients recruited in the “real world” observational study at the University of Southampton (NIHR/UKCRN ID: 31076, CI F.Forconi) following written informed consent in accordance with Ethics Committee approvals (UK National Research Ethics Service number 19/WM/0262) and the Declaration of Helsinki. Diagnosis of CLL was according to the 2008 International Workshop on CLL (IWCLL2008)/National Cancer Institute (NCI) criteria (26). Diagnosis was confirmed by a flow cytometry “Matutes score” >3 in all cases. Phenotypic and immunogenetic characteristics (tumour *IGHV* usage and mutational status) were determined as previously described (27). CLL patient, HCC patient and healthy donor PBMCs were cryopreserved in liquid nitrogen.

Human liver cancer cell lines (HepG2 and PLC/PRF5) were maintained in DMEM (Gibco) supplemented with 1% penicillin-streptomycin (Life Technologies) and 10% FBS (Sigma). PBMC, Raji cells and the HLA-null 721.221 B cell lymphoblastoid cell line were cultured in RPMI 1640 (Gibco) supplemented with 1% penicillin-streptomycin and 10% FBS (R10). Status of HLA-C expression of these cells lines has previously been reported (25).

### 2.2 *Ex vivo* NK cell expansion

NK cells were isolated from healthy donor PBMCs using the Miltenyi NK isolation kit (130-092-657) with ~95% purity (**Supplementary Figure 1**). NK cells were cultured in expansion media consisting of NK MACS medium (Miltenyi Biotech) supplemented with 1% NK supplement (Miltenyi Biotech), 5% human AB serum (Sigma-Aldrich) and 500 IU/mL IL-2 (Miltenyi Biotech) for 14-21 days at 37°C before use in functional assays as indicated in the figure legends. Alternatively, NK cells were cultured in 10 ng/mL IL-12, 20 ng/mL IL-15 and 50 ng/mL IL-18 (R&D Systems) for 16 hours before the media was replaced with expansion media as above. After the first 5 days, expansion media was replenished every 2-3 days to maintain cells at 0.4-0.5 x 10^6^ cells/mL.

### 2.3 NK cell activation assays

Healthy donor PBMCs stimulated overnight in 1 ng/mL IL-15 (R&D Systems), or NK cells expanded for 14-21 days were used as effector cells as indicated in the figure legends. The tumour-targeting antibodies cetuximab (human IgG1, in-house), avelumab (human IgG1, Southampton General Hospital (SGH) pharmacy), rituximab (human IgG1, in-house), obinutuzumab (human IgG1, SGH pharmacy) or isotype control (human IgG1, ChiLob7-4, in-house) were added to the target cells as indicated for 20 minutes before being washed twice with media to remove unbound antibody. Effector cells were then co-incubated with target cells at the indicated E:T ratios for 4 hours at 37°C/5% CO_2_ in a 96 well round bottomed plate. Samples were then stained with 0.17 μg/mL anti-CD107a-e660 (eBioscience) and after 1 hour of co-culture, Golgistop (BD Biosciences) was added for a further 3 hours. Samples were then stained for surface markers with the following antibodies in FACS buffer (PBS, 1% BSA, 0.05% Sodium Azide) for 30 minutes at 4°C: CD3-PerCP (UCHT1, Biolegend), CD56-PE/Cy7 (HCD56, Biolegend), KIR2DL3/L2/S2-PE (CH-L, BD Biosiences) and KIR2DL3/L2-FITC (REA147, Miltenyi Biotech). For assessment of IFNγ expression, cells were permeabilised with BD Cytofix/Cytoperm (BD Biosciences) and stained with IFNγ-BV421 (Biolegend) following the manufacturer’s protocol and analysed using a BD FACS Aria II and FlowJo V10.8.1 software. Data shown in graphs were calculated by subtracting CD107a or IFNγ readings of the no target control from data with targets.

HCC and CLL patient PBMCs were used in assays on the day of thaw and were not cultured in IL-15 due to poor long-term sample viability. For experiments using KIR2DS2+ CLL donors, samples with ≥6% CD5-CD19- lymphocytes were selected and CLL donor PBMCs (250,000 cells/100 μL) were labelled with 0.17 μg/mL anti-CD107a-AF660 and incubated with indicated tumour-targeting antibodies for one hour prior to addition of Golgistop for a further 3 hours. PBMC were stained with 1 μg/mL DAPI and DAPI+ (dead) cells were removed from the analysis. This assay set-up lacked a no target control, therefore data presented in graphs are the raw CD107a and IFNγ expression values.

To assess direct cytotoxicity of the different NK cell subsets, KIR2DS2^high^, KIR2DL3/L2^high^, and KIR2DL3/L2/S2− CD56^dim^ CD3− healthy human cells were sorted using a BD FACS Aria II using the surface staining protocol described above. NK cells were then cultured overnight in R10 supplemented with 1ng/ml IL-15. 721.221 cells were stained with Cell Trace™ Violet Cell Proliferation Kit (Invitrogen) and were then co-cultured with the sorted NK cells at an effector:target (E:T) ratio of either 1:1 or 3:1 for 4 h at 37°C. After co-culture, cells were stained with propidium iodide (Invitrogen) and lysis of 721.221 cells was assessed on a BD FACS Aria II (BD Biosciences) using FACSDiva software (BD Biosciences) and analysed with FlowJo v10.7.1 (BD Biosciences).

### 2.4 Phenotyping by flow cytometry

To assess CD16 expression on patient samples, cells were stained with CD16-APC (3G8, Biolegend) in FACS buffer for 30 minutes at 4°C. To assess expression of activating receptors on the surface of NK cells before and after expansion, cells were stained with NKp30-PerCP (9E2, Biolegend), NKp46-APC (P30-15, Biolegend), NKG2D-APC/Cy7 (1D11, Biolegend) and CD57-APC (HNK-1, Biolegend) in FACS buffer for 30 minutes at 4°C. All samples were analysed using a BD FACS Aria II and FlowJo V10.8.1 software.

### 2.5 HLA-C and KIR genotyping

Full-length PCR amplification of HLA-C was performed with VeriFi DNA polymerase (PCR Biosystems, UK) using in-house primers and protocols at the Anthony Nolan Research Institute (ANRI). Amplicon size and concentration were determined using a Fragment Analyzer (Agilent). Sequencing libraries were generated using PacBio TPK3 (Pacific Biosciences) as per manufacturer’s instructions and then sequenced by a Pacific Biosciences Sequel machine (28). Analysis was performed using an in-house HLA genotyping pipeline at ANRI as described in (29, 30). KIR genotype was confirmed by polymerase chain reaction sequence-specific primer (PCR-SSP) reactions detailed in (31).

### 2.6 Statistical analysis

Statistical significance was determined using GraphPad PRISM software (version 10.0.2). One-way ANOVA was used to compare more than 2 groups with one independent variable and two-way ANOVA was used to compare more than 2 groups with 2 independent variables. Geisser-Greenhouse correction for unequal variability and Dunnett’s correction for multiple comparisons were applied to two-way ANOVA analyses. Data was considered statistically significant at p<0.05.

## 3 Results

### 3.1 KIR2DS2+ NK cells from healthy donors and cancer patients display enhanced activation in response to tumour-targeting antibodies

**Figure 1:**
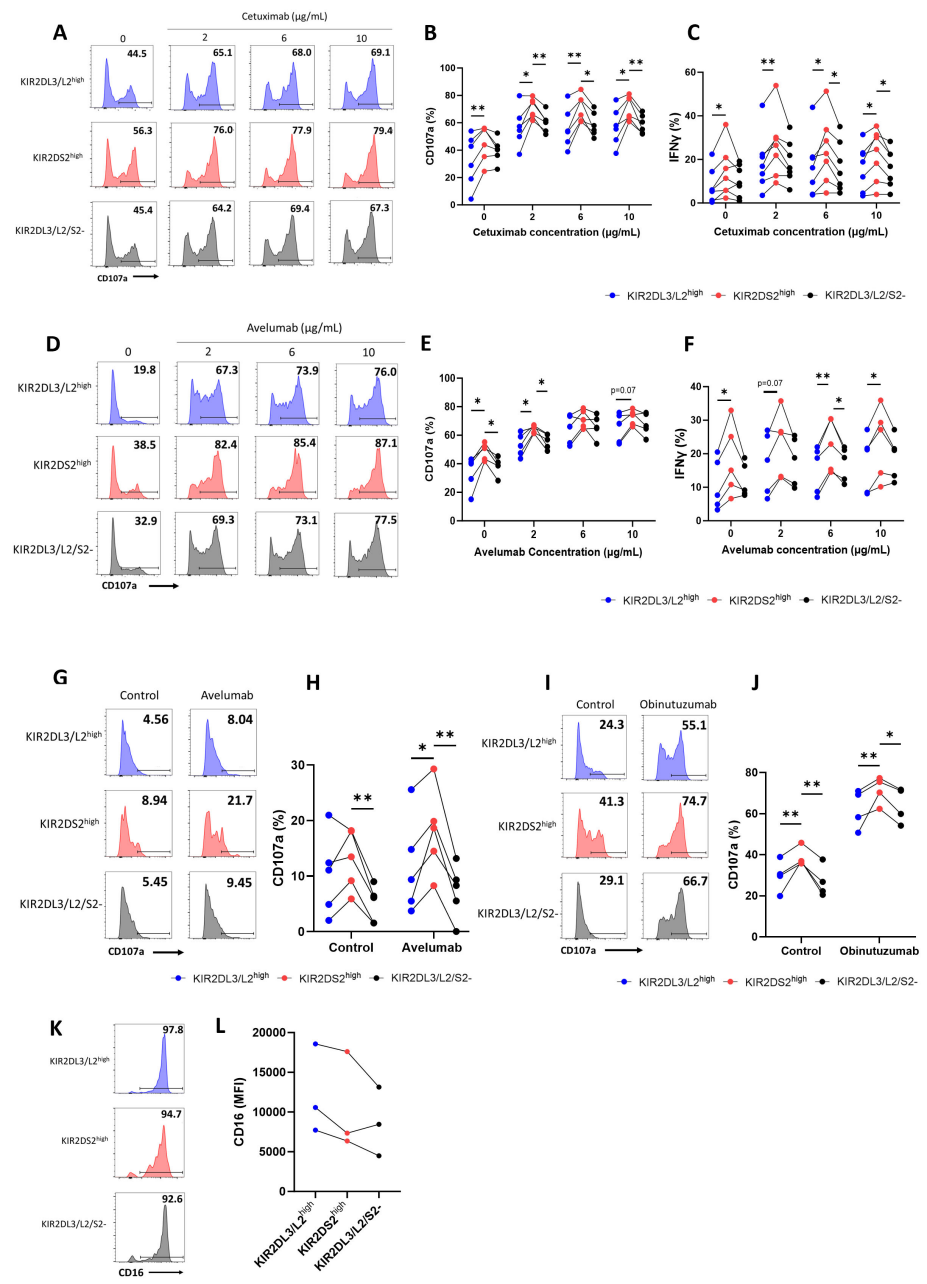
KIR2DS2^high^ NK cells exhibit enhanced activation against hepatocellular carcinoma cells in response to tumour-targeting antibodies. PLC/PRF/5 **(A-C)** and HepG2 **(D-F)** cells were incubated with the indicated concentrations of cetuximab or avelumab, respectively, before co-culture with 1 ng/mL IL-15-primed healthy donor NK cells for 4 hours at either a 10:1 (CD107a) or 5:1 (IFNγ) effector:target (E:T) ratio. CD107a and IFNγ staining on the KIR2DL3/L2^high^, KIR2DS2^high^ or KIR2DL3/L2/S2- NK cell subpopulations were measured by flow cytometry. Representative data are shown in A and D and summarised data shown for CD107a **(B, E)** and IFNγ **(C, F)** (n=5-7). HepG2 **(G, H)** or 721.221 **(I, J)** cells were incubated with the indicated antibodies before co-culture with NK cells from hepatocellular carcinoma (HCC) patients for 4 hours at a 5:1 E:T ratio. CD107a expression on NK cells was measured by flow cytometry. Representative data shown in G and I and summarised data shown in H and J (n=4-5). Data shown in graphs were calculated by subtracting CD107a or IFNγ readings of the no target control from data with targets. **(K, L)** CD16 expression on KIR2DL3/L2^high^, KIR2DS2^high^ or KIR2DL3/L2/S2- NK cell subpopulations from HCC patients. Representative data with annotated % CD16+ cells shown in K and CD16 MFI values of 3 donors shown in L. Analysed by two-way or one-way ANOVA using Graphpad PRISM. P<0.05*, p<0.01**, S.D., standard deviation.

HCC has an extremely poor survival rate (32) and NK cell therapies represent a promising novel treatment approach that are currently under clinical evaluation (NCT04162158, NCT02008929, NCT05040438). KIR2DS2^high^ NK cells isolated from healthy donors have previously been shown to possess enhanced activation against lymphoma cells in the presence of anti-CD20 antibodies (25) in comparison to KIR2DS2- NK cells, however, it is not known whether this enhanced activity is also present for KIR2DS2^high^ NK cells in response to antibodies directed against solid tumour targets. Both PD-L1 and EGFR can be upregulated on the surface of HCC tumour cells (33) and antibodies targeting the PD-1: PD-L1 axis are approved in HCC (34). We therefore assessed NK activation against HCC cell lines in the presence of the anti-EGFR antibody cetuximab and the anti-PD-L1 antibody avelumab, both of which are known to induce ADCC against target cells (35, 36). HepG2 and PLC/PRF/5 HCC tumour cells were incubated with the indicated concentrations of avelumab or cetuximab as indicated prior to co-culture with healthy donor NK cells that had been primed overnight with 1 ng/mL IL-15. All healthy donors used in this study had at least one copy of HLA-C1 (**Table 1**) and their NK cells were therefore educated through KIR2DL3/L2/S2. Using a previously published flow cytometry panel to separate NK cells based on KIR2DL3/L2/S2 expression (**Supplementary Figure 2**) (37), KIR2DS2^high^ NK cells demonstrated significantly higher degranulation against HCC cells compared with KIR2DL3/L2^high^ and KIR2DL3/L2/S2- NK cells alone and in combination with all concentrations of cetuximab tested (p<0.01 or p<0.05) (**Figures 1A, B**). Induction of IFNγ was also higher in the KIR2DS2^high^ population relative to the other NK cell subsets (**Figure 1C**). Importantly, CD107a and IFNγ expression were comparable between the NK cell subsets in the absence of target cells (**Supplementary Figure 2**). Furthermore, KIR2DS2^high^ NK cells demonstrated enhanced degranulation and IFNγ expression against HCC cells alone and in combination with avelumab compared to KIR2DL3/L2^high^ and KIR2DL3/L2/S2- NK cells (p<0.01 or p<0.05, respectively) (**Figures 1D-F**). At the higher concentrations of avelumab used, which were based on the achievable serum concentrations in patients (38), degranulation was saturated (**Figure 1E**), potentially masking the differences in ADCC between the NK cell subsets. Furthermore, in accordance with their enhanced activation status, KIR2DS2^high^ NK cells induced greater lysis of target cells compared to KIR2DL3/L2^high^ and KIR2DL3/L2/S2- NK cells (**Supplementary Figure 3**).

**Table 1:** HLA-C genotyping of healthy donors, HCC and CLL patients.

	HLA-C alleles		HLA-C allotypes
Healthy donors			
**1**	C*04:01:01	C*07:01:01	C1C2
**2**	C*05:01:01	C*07:01:01	C1C2
**3**	C*04:01:01	C*07:01:01	C1C2
**4**	C*07:04:01	C*08:02:01	C1C1
**5**	C*12:03:01	C*14:02:01	C1C1
**6**	C*06:02:01	C*12:03:01	C1C2
**7**	C*07:01:01	C*12:03:01	C1C1
**8**	C*07:01:01	C*07:01:01	C1C1
**9**	C*01:02:01	C*15:02:01	C1C2
HCC patients			
**14**	NA	NA	NA
**19**	C*05:01:01	C*06:02:01	C2C2
**22**	C*07:01:01	C*07:02:01	C1C1
**23**	C*07:01:01	C*07:02:01	C1C1
**24**	NA	NA	NA
CLL patients			
**656**	C*03:04:01	C*07:02:01	C1C1
**1447**	C*07:02:01	C*07:04:01	C1C1
**892**	C*07:01:01	C*07:01:01	C1C1
**484**	C*02:02:02	C*07:04:01	C1C2
**1038**	C*04:01:01	C*06:02:01	C2C2
**469**	C*02:02:02	C*05:01:01	C2C2

NA, not available.

Because therapeutic strategies including tumour-targeting mAbs, cytokines and NK cell engagers (NKCEs) rely on the effector functions of patient NK cells, we sought to evaluate whether the enhanced function associated with KIR2DS2 was also evident in NK cells isolated from patients with cancer. PBMCs isolated from HCC patients were co-cultured with target cell lines, in the presence or absence of the indicated ADCC-inducing antibodies. Against HepG2 cells, KIR2DS2^high^ NK cells derived from the peripheral blood of HCC patients (n=5) demonstrated enhanced activation compared to KIR2DL3/L2/S2- NK cells (p<0.01) in both the presence and absence of avelumab (**Figures 1G, H**). Significantly higher activation was also observed compared to KIR2DL3/L2^high^ NK cells (p<0.05) in the presence of avelumab (**Figure 1H**). In addition, KIR2DS2^high^ NK cells from HCC patients had enhanced activation against HLA-deficient 721.221 target cells both in the absence and presence of the type II anti-CD20 mAb obinutuzumab (p<0.01) compared to KIR2DL3/L2^high^ and KIR2DL3/L2/S2- NK cells (**Figures 1I, J**). This indicates that the enhanced activation associated with KIR2DS2 is not solely due to less inhibitory signalling compared to KIR2DL3/L2^high^ cells or binding of KIR2DS2 to HLA-C. This is in accordance with data previously observed using healthy donor derived NK cells (25). Of the three HCC patients tested, CD16 expression was not significantly higher on KIR2DS2^high^ NK cells compared to KIR2DL3/L2^high^ and KIR2DL3/L2/S2- NK cells (**Figures 1K, L**), indicating that the enhanced ADCC of the KIR2DS2^high^ population was not simply due to greater expression of CD16.

**Figure 2:**
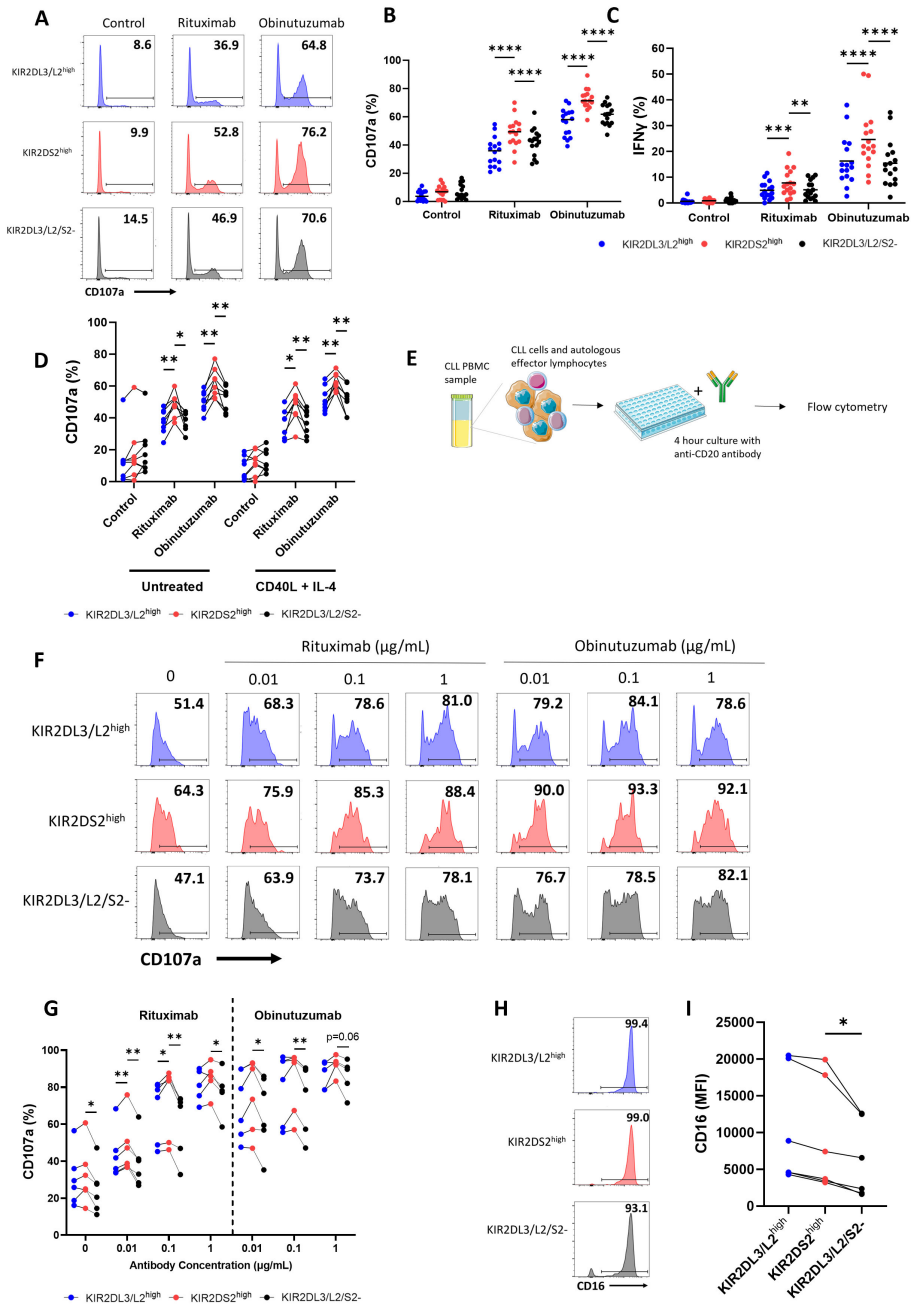
Autologous and allogeneic KIR2DS2^high^ NK cells exhibit enhanced activation against chronic lymphocytic leukaemia cells in response to anti-CD20 antibodies. Primary chronic lymphocytic leukaemia (CLL) samples with >90% tumour were incubated with rituximab (10 μg/mL), obinutuzumab (1 μg/mL) or control antibody (10 μg/mL) for 20 mins before co-culture with 1 ng/mL IL-15-primed healthy donor NK cells for 4 hours at a 5:1 E:T ratio. CD107a and IFNγ expression on the KIR2DL3/L2^high^, KIR2DS2^high^ or KIR2DL3/L2/S2- NK cell subpopulations were measured by flow cytometry. Representative data are shown in **(A)** with summarised data shown for CD107a **(B)** and IFNγ **(C)** (n=16). **(D)** Primary CLL cells were incubated with or without CD40L (300 ng/mL) and IL-4 (10 ng/mL) for 24 hours. Cells were incubated with antibodies as above before co-culture with IL-15-primed healthy donor NK cells at a 5:1 E:T as above. CD107a expression of the different NK cell subsets was measured by flow cytometry (n=6). Data shown in graphs were calculated by subtracting CD107a or IFNγ readings of the no target control from data with targets. **(E)** KIR2DS2+ CLL patient PBMC samples with ≥6% healthy CD5-CD19- lymphocytes were incubated with rituximab or obinutuzumab at the indicated concentrations, or isotype control (1 μg/mL) for a total of 4 hours before CD107a expression on NK cells was measured by flow cytometry. Representative data is shown in **(F)** and summarised data in **(G)** (n=5-6). **(H, I)** CD16 expression on KIR2DL3/L2^high^, KIR2DS2^high^ or KIR2DL3/L2/S2- NK cell subpopulations from CLL patients were measured by flow cytometry. Representative data with annotated % CD16+ cells shown in **(H)** and CD16 MFI values of 6 donors shown in **(I)** Analysed by two-way or one-way ANOVA using Graphpad PRISM. P<0.05*, p<0.01**, p<0.001***, p<0.0001****, S.D. = standard deviation. Parts of this figure were generated using Servier Medical Art images licensed under a Creative Commons Attribution 3.0 Unported License (https://creativecommons.org/licenses/by/3.0/).

To determine the antibody-dependent activation of KIR2DS2^high^ NK cells against primary tumour samples, we used peripheral blood derived chronic lymphocytic leukaemia (CLL) cells as targets in combination with the clinically relevant anti-CD20 antibodies rituximab and obinutuzumab. Obinutuzumab is known to induce ADCC more potently than rituximab due to its enhanced affinity for CD16 (38). In the presence of these antibodies, KIR2DS2^high^ NK cells from healthy donors primed overnight with 1 ng/mL IL-15 showed significantly higher CD107a (p<0.0001) (**Figures 2A, B**) and IFNγ (p<0.0001, p<0.001 or p<0.01) (**Figure 2C**) expression compared to the other NK cell subsets in accordance with previous reports against lymphoma cell lines (25). Furthermore, as the lymph nodes are a key tissue site for CLL proliferation and drug resistance in patients (39), we tested the activation of the NK cells in the presence of signals mimicking the lymph node microenvironment (CD40L and IL-4). KIR2DS2^high^ NK cells retained enhanced activation in the presence of CD40L and IL-4 (p<0.01 or p<0.05) (**Figure 2D**).

Subsequently, we assessed the function of KIR2DS2^high^ NK cells from PBMC isolated from CLL patients against their own autologous tumour cells (**Figure 2E**). Six CLL patients positive for KIR2DS2 expression with sufficient (≥6%) healthy CD5-CD19- lymphocytes were identified by flow cytometry (37). Patient PBMC were incubated with rituximab or obinutuzumab as indicated before assessment of NK cell degranulation by flow cytometry in the CD3-CD56+ lymphocyte population. KIR2DS2^high^ NK cells from CLL patients showed significantly enhanced activation in the absence of anti-CD20 mAb compared to KIR2DL3/L2/S2- (p<0.05) but not KIR2DL3/L2^high^ NK cells (**Figures 2F, G**). In the presence of rituximab, enhanced activation of the KIR2DS2^high^ population was evident compared with both KIR2DL3/L2/S2- (p<0.01 for 0.01 and 0.1 μg/mL, p<0.05 for 1 μg/mL) and KIR2DL3/L2^high^ NK cells (p<0.01 for 0.01 μg/mL, p<0.05 for 0.1 μg/mL) (**Figure 2G**). In the presence of obinutuzumab, KIR2DS2^high^ NK cells showed enhanced activation compared to KIR2DL3/L2/S2- cells (p<0.05 for 0.01 μg/mL, p<0.01 for 0.1 ug/mL), but did not reach statistical significance compared to KIR2DL3/L2^high^ NK cells (**Figure 2G**). Finally, similar to HCC patient NK cells, levels of CD16 expression were comparable between KIR2DS2^high^ and KIR2DL2/L3^high^ NK cells, however CD16 was significantly higher on KIR2DS2^high^ cells compared to KIR2DL3/L2/S2- cells (p<0.05) (**Figures 2H, I**). These data indicate that KIR2DS2 expression is associated with a population of NK cells in cancer patients that have superior activation against target cells both in the presence and absence of tumour-targeting antibodies.

### 3.2 Enhanced activation of KIR2DS2+ NK cells from healthy donors is lost after *ex vivo* expansion

**Figure 3:**
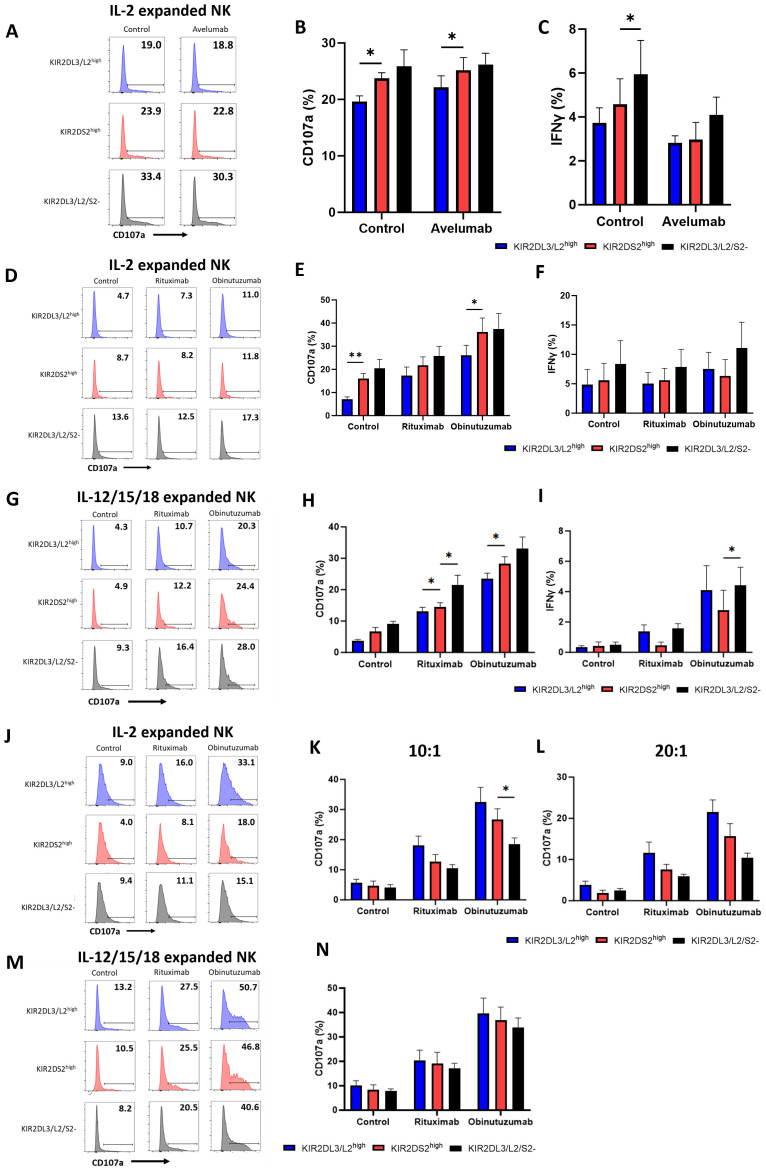
The superior effector function of KIR2DS2^high^ NK cells is lost following *ex vivo* expansion. **(A-C)** NK cells were isolated from healthy donor PBMCs and cultured in NK MACS medium with IL-2 (expansion media) for 14-21 days. Expansion media was added every 2-3 days. HepG2 cells were incubated with avelumab or control antibody for 20 mins before co-culture with expanded NK cells at a 5:1 E:T for 4 hours. CD107a and IFNγ expression of NK cells was measured by flow cytometry. Representative data are shown in A and data is summarised as mean ± SEM for CD107a **(B)** and IFNγ **(C)** (n=5-6). **(D-M)** NK cells were expanded in either IL-2 alone as above or IL-12/15/18 for 16 hours as indicated followed by expansion media. **(D-I)** Raji cells were incubated with the indicated antibodies for 20 mins before co-culture with expanded NK cells at a 0.1:1 E:T for 4 hours. CD107a and IFNγ expression on NK cells was measured by flow cytometry. Representative plots for NK activation against Raji cells shown in **(D, G)** and data is summarised as mean ± SEM for CD107a **(E, H)** and IFNγ **(F, I)** (n=4-6). **(J-N)** 721.221 cells were incubated with the indicated antibodies for 20 minutes before co-culture with IL-2 expanded NK cells at a 10:1 or 20:1 E:T ratio or IL-12/15/18 expanded NK cells at a 10:1 E:T ratio as indicated for 4 hours. CD107a expression was measured by flow cytometry. Representative data is shown in **(J, M)** and data is summarised as mean ± SEM in K, L and N (n=4). Data shown in graphs were calculated by subtracting CD107a or IFNγ readings of the no target control from data with targets. Analysed by two-way ANOVA using Graphpad PRISM. P<0.05*, p<0.01**, SEM, standard error of mean.

*Ex vivo* expansion is required for adoptive NK cell therapies. We therefore assessed whether the enhanced activation of KIR2DS2+ NK cells was retained following expansion *ex vivo* and could therefore potentially be used to select a population of NK cells with enhanced anti-cancer activity. NK cells were isolated from the peripheral blood of healthy volunteers, mimicking the source of NK cells in allogeneic adoptive transfer strategies (7). The NK cells were then expanded in Miltenyi Biotech NK expansion medium with human IL-2 for 14-21 days (**Supplementary Figure 4**). The Miltenyi Biotech NK expansion method has recently been shown to have potential for the clinical production of expanded NK cells (40). Expanded NK cells were then co-cultured with HepG2 cells for 4 hours in combination with avelumab (**Figure 3A**). After expansion, KIR2DS2^high^ NK cells lost their enhanced activation relative to the KIR2DL3/L2/S2- subset but not the KIR2DL3/L2^high^ subset as measured by both degranulation (p<0.05 KIR2DS2^high^ vs KIR2DL3/L2^high^) and IFNγ (p<0.05 KIR2DS2^high^ vs KIR2DL3/L2/S2-) expression (**Figures 3A-C**). In accordance with the data against HepG2 target cells, KIR2DS2^high^ NK cells also lost their enhanced activation over the KIR2DL3/L2/S2- but not the KIR2DL3/L2^high^ (p<0.01 for control, p<0.05 for obinutuzumab) subset of NK cells against CD20+ malignant B cells (Raji) following *ex vivo* expansion (**Figures 3D-F**). Expression of the activating receptors NKp30, NKp46 and NKG2D was significantly increased on the surface of each subset of NK cells after *ex vivo* expansion in IL-2 for 14 days compared to day 0 (p<0.0001 for NKp30 and NKG2D, p<0.001 for NKp46), although there were no significant differences in expression of these receptors between NK cell subsets on day 0 or day 14 (**Supplementary Figures 5A-C**). In addition, CD57 was consistently higher on KIR2DS2^high^ NK cells compared to KIR2DL3/L2/S2- NK cells and decreased on all three NK cell subsets after expansion in IL-2 (**Supplementary Figure 5D**). To determine if the loss of enhanced activation in the KIR2DS2^high^ NK cell subset also occurred with other methods of NK expansion, we tested the effect of an expansion method utilizing IL-12/15/18. The IL-12/15/18 cytokine cocktail has been shown to generate cytokine-induced memory-like NK cells and is currently in clinical trials for patients with cancer (41, 42). In accordance with the results from the previous experiments, KIR2DS2^high^ NK cells expanded in IL-12/15/18 did not have higher activation compared to the KIR2DL3/L2/S2- subset in combination with rituximab and obinutuzumab (**Figures 3G-I**).

To determine whether this effect was due to inhibitory KIR: HLA interactions, we tested *ex vivo* expanded NK cell activation against the HLA-deficient B cell lymphoblastoid cell line 721.221. In this setting, KIR2DL3/L2^high^ NK cells showed a trend for higher activation compared to KIR2DS2^high^ NK cells and KIR2DL3/L2/S2- NK cells at both a 10:1 and 20:1 E:T ratio (**Figures 3J-L**). Similar results were obtained for KIR2DS2^high^ NK cells expanded in IL-12/15/18 (**Figures 3M, N**). Overall, these results demonstrate that the superior effector function associated with KIR2DS2+ NK cells may not be applicable in an adoptive transfer setting that requires *ex vivo* expansion of NK cells.

## 4 Discussion

Immunogenetic and functional studies have identified that the activating receptor KIR2DS2 is associated with enhanced NK cell function against cancer cells. However, it was unclear whether KIR2DS2+ NK cells retain their enhanced functional state in cancer patients or following *ex vivo* expansion protocols which are required for NK cell therapies such as adoptive transfer and CAR-NK cells. Here, we demonstrate that the superior effector function associated with KIR2DS2 expression in combination with tumour-targeting antibodies is retained in cancer patients, however this effect is lost following *ex vivo* expansion. This indicates that KIR2DS2 may be an attractive therapeutic target for the selective activation of a highly active NK cell subset in cancer patients but may be less important for adoptive transfer strategies utilising *ex vivo* expanded NK cells from peripheral blood.

NK cells can become dysfunctional in patients with cancer (5) with reduced expression of NK cell activating receptors such as NKp30, NKp46 and NKG2D and upregulation of inhibitory checkpoint receptors such as TIGIT (43). In this study, enhanced function of KIR2DS2-expressing NK cells isolated from healthy donors and cancer patients was evident against liver cancer cell lines and primary CLL samples in the presence of clinically relevant tumour-targeting antibodies. KIR2DS2 expression has previously been associated with beneficial response to antibodies in neuroblastoma and small molecule inhibitors in CML (44, 45) and our *ex vivo* data indicates that beneficial effects may also been seen in HCC and B cell malignancies with tumour-targeting antibodies, however this remains to be assessed *in vivo*. The high sequence homology between the activating and inhibitory KIR limits the analysis of the association between KIR2DS2 and survival in publicly available cancer patient RNA-sequencing datasets. In addition, enhanced activation of the KIR2DS2^high^ population was also evident in the absence of tumour-targeting antibodies with HCC patient NK cells, in accordance with previous reports with healthy donors (25), and we now show that the enhanced activation is also retained in the presence of avelumab and cetuximab. In accordance with the functional data presented in this study, single-cell RNA-sequencing analysis has previously shown that KIR2DS2 is associated with high expression of NK cell cytotoxicity-related genes including GNLY, NKG7 and granzymes (25). This indicates that KIR2DS2^high^ NK cells are transcriptionally primed for enhanced cytotoxicity in the presence and absence of CD16 engagement via tumour-targeting antibodies. Although we did not perform RNA-sequencing on the patient samples in this study, the functional data is in agreement with these previous findings.

These data indicate that KIR2DS2 could be a promising target to potentiate NK cell function in cancer patients via a novel agonistic antibody or NKCE, as has been developed for other receptors including NKp46 and NKG2D (46). Indeed, the ligation of KIR2DS2 induces NK cell activation via recruitment of ZAP-70 and Syk, leading to phosphorylation and activation of downstream targets and signalling pathways (47). The >98% sequence homology in the extracellular domains of KIR2DS2 and the inhibitory KIRs KIR2DL2 and KIR2DL3 has however been a limiting factor in this approach to date (13). Generation of a selective KIR2DS2 antibody remains a possibility however, as the generation of antibodies able to distinguish between proteins with high homology in the external domains has been previously described (48). Current phage display technologies are also allowing for the development of antibodies with exquisite specificity and high affinity (49, 50). An alternative approach which may have utility is the stimulation of KIR2DS2 via the natural viral derived peptide ligands of KIR2DS2 in conjunction with HLA-C (37, 51).

As well as directly targeting NK cells in patients, NK cells from healthy donors are being assessed in adoptive transfer therapeutic strategies. To achieve sufficient cell numbers of donor-derived NK cells for a viable off-the-shelf allogeneic product, it is necessary to expand NK cells *ex vivo*. Selection of NK cell donors based on the KIR haplotype B, which contains a variable number of activating KIR and can include KIR2DS2, has already entered clinical trials for non-Hodgkin lymphoma (NCT04673617) (52). Following *ex vivo* expansion in IL-2 or IL-12/15/18 in our study, KIR2DS2^high^ NK cells lost their superior activation against HLA-expressing targets compared to NK cells lacking KIR2DL3/L2/S2 expression but not compared to the inhibitory KIR2DL3/L2^high^ population. However, the superior activation against KIR2DL3/L2^high^ cells was likely due to less inhibitory KIR signalling in the KIR2DS2^high^ population (37) because the enhanced reactivity was lost against HLA-null target cells. This contrasts with previous work which demonstrated that healthy donor KIR2DS2^high^ NK cells primed with IL-15 overnight have enhanced activity in the absence of HLA expression on target cells (25). This therefore indicates that KIR2DS2+ NK cells from peripheral blood lose their association with enhanced functional capacity following expansion. *Ex vivo* expansion in IL-2 has previously been demonstrated to alter NK cell function, receptor expression and gene expression (53–55) and in accordance with this, we identified significantly increased expression of the activating receptors NKp30, NKp46 and NKG2D in the KIR2DS2^high^, KIR2DL3/L2^high^ and KIR2DL3/L2/S2- NK cell subpopulations tested. This may therefore override the native capacity for KIR2DS2+ NK cells to possess enhanced effector functions evident in freshly isolated cells. Expanding NK cells from other sources such as cord blood or utilising other NK expansion methods (7) may retain the enhanced activation of KIR2DS2, however this remains to be tested. Overall, these data indicate that whilst KIR2DS2 is an attractive target for *in vivo* targeted NK cell immunotherapeutic strategies, this functional advantage is lost following *ex vivo* expansion.

## Data Availability

The raw data supporting the conclusions of this article will be made available by the authors, without undue reservation.
