# Supplementary material for: KIR2DS2+ NK cells in cancer patients demonstrate high activation in response to tumour-targeting antibodies
Source: Front Oncol. 2024 Sep 2;14:1404051. doi: 10.3389/fonc.2024.1404051 (PMC11402612; doi:10.3389/fonc.2024.1404051)
Supplement: Supplementary file 1 [file DataSheet1.docx]

| **Supplementary Table 1: HCC patient information.**  NAFLD = Non-alcoholic fatty liver disease. N/A = not available. | | | |
| --- | --- | --- | --- |
| **Donor** | **Gender** | **Age** | **Disease** |
| 14 | M | 79 | Alcohol/NAFLD |
| 19 | M | 52 | NAFLD |
| 22 | M | 77 | N/A |
| 23 | F | 58 | NAFLD |
| 24 | F | 68 | Alcohol |

| **Supplementary Table 2: CLL patient information.**  IGHV = Immunoglobulin heavy chain variable region. M-CLL = Mutated IGHV. U-CLL = Unmutated IGHV. N/A = not available. | | | | | | |
| --- | --- | --- | --- | --- | --- | --- |
| **Patient number** | **Gender** | **Age at diagnosis** | **Binet Stage** | **Rai stage** | **IGHV** | **Tumour %** |
| 656 | Female | 76 | B | 1 | M-CLL | 68.3 |
| 1447 | N/A | 74 | A | 1 | M-CLL | 56 |
| 892 | Male | 65 | A | 1 | M-CLL | 54 |
| 484B | Male | 46 | A | 0 | M-CLL | 67.3 |
| 1038 | Male | 83 | A | 0 | M-CLL | 75 |
| 469C | Male | 67 | A | 2 | N/A | 61.2 |
| 1459 | Male | 66 | C | 4 | U-CLL | 95.7 |
| 1414 | Male | 67 | A | 0 | U-CLL | 96.9 |
| 809 | Male | 70 | A | 1 | U-CLL | 89.4 |
| 1003 | Male | 51 | A | 1 | U-CLL | 95.6 |
| 368C | Male | 52 | A | N/A | U-CLL | 93.0 |
| 1451 | N/A | 70 | A | 1 | M-CLL | 94.0 |
| 1220 | Male | 56 | A | N/A | M-CLL | 86.0 |
| 1322 | Male | 84 | C | 4 | M-CLL | 90.3 |
| 1358 | Male | 74 | A | 2 | M-CLL | 94.7 |
| 1352 | Female | 67 | B | 1 | M-CLL | 88.3 |
| 1373 | Male | 60 | N/A | N/A | M-CLL | 92.2 |

| 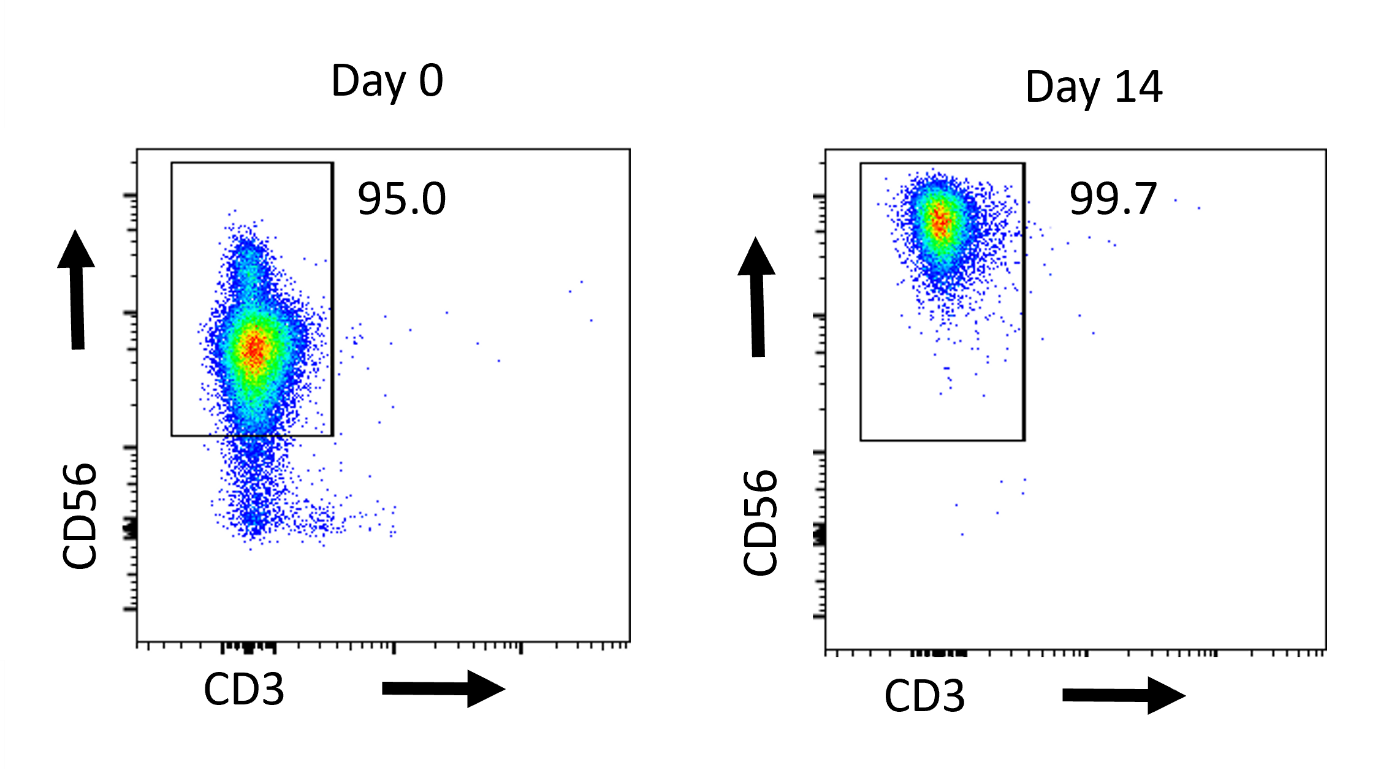 |
| --- |
| **Supplementary Figure 1: Representative purity of NK cells at day 0 and day 14 of expansion.**  Primary human NK cells NK cells (CD3-CD56+) were isolated from healthy donor bulk PBMC using the Miltenyi Biotech human NK cell isolation kit. NK cells were then expanded *ex vivo* using Miltenyi Biotech NK cell MACS medium with IL-2 (expansion media). Expansion media was added every 2-3 days. Purity was checked on day 0 and day 14 by flow cytometry. |

| 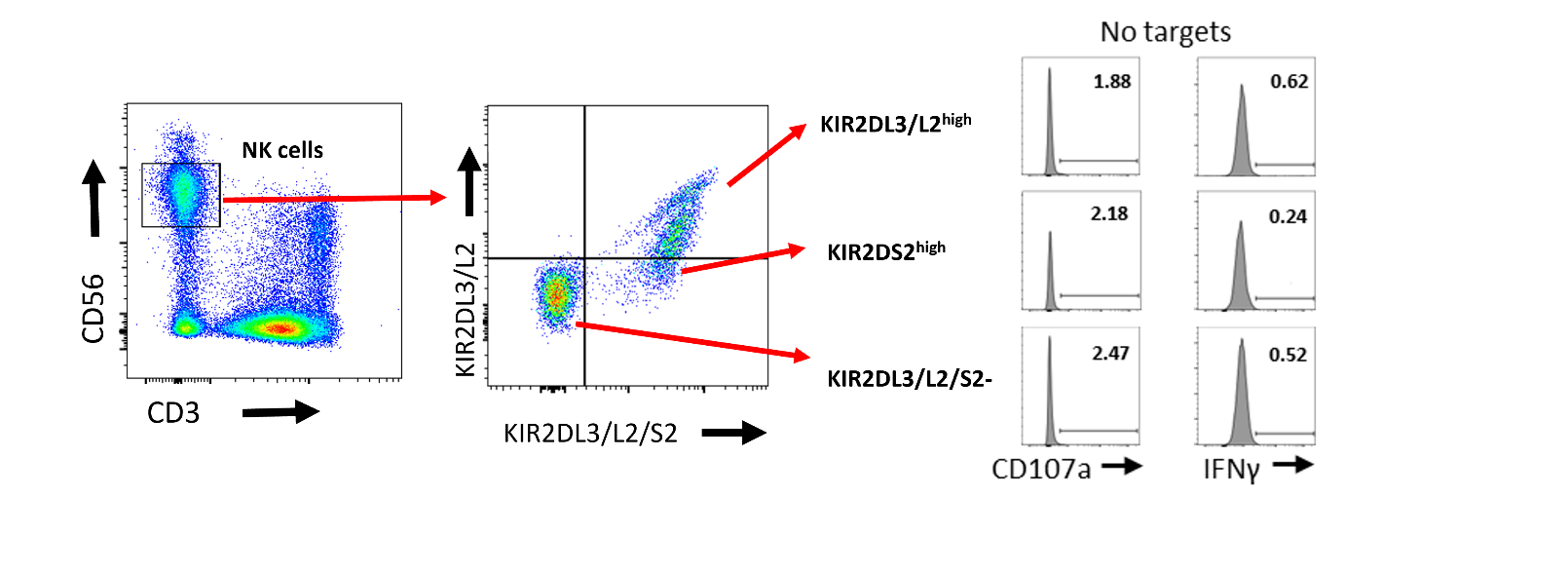 |
| --- |
| **Supplementary Figure 2: Gating strategy for identifying KIR2DS2^high^, KIR2DL3/L2^high^ and KIR2DL3/L2/S2- NK cells by flow cytometry.**  CD3-CD56^dim^ NK cells were separated into KIR2DS2^high^, KIR2DL3/L2^high^ or KIR2DL3/L2/S2- subpopulations by flow cytometry using antibodies against KIR2DL3/L2 (REA147) and KIR2DL3/L2/S2 (CH-L) combined as indicated. Representative plots for CD107a and IFNγ expression within the three NK cell subsets in the absence of target cells are shown. |

| 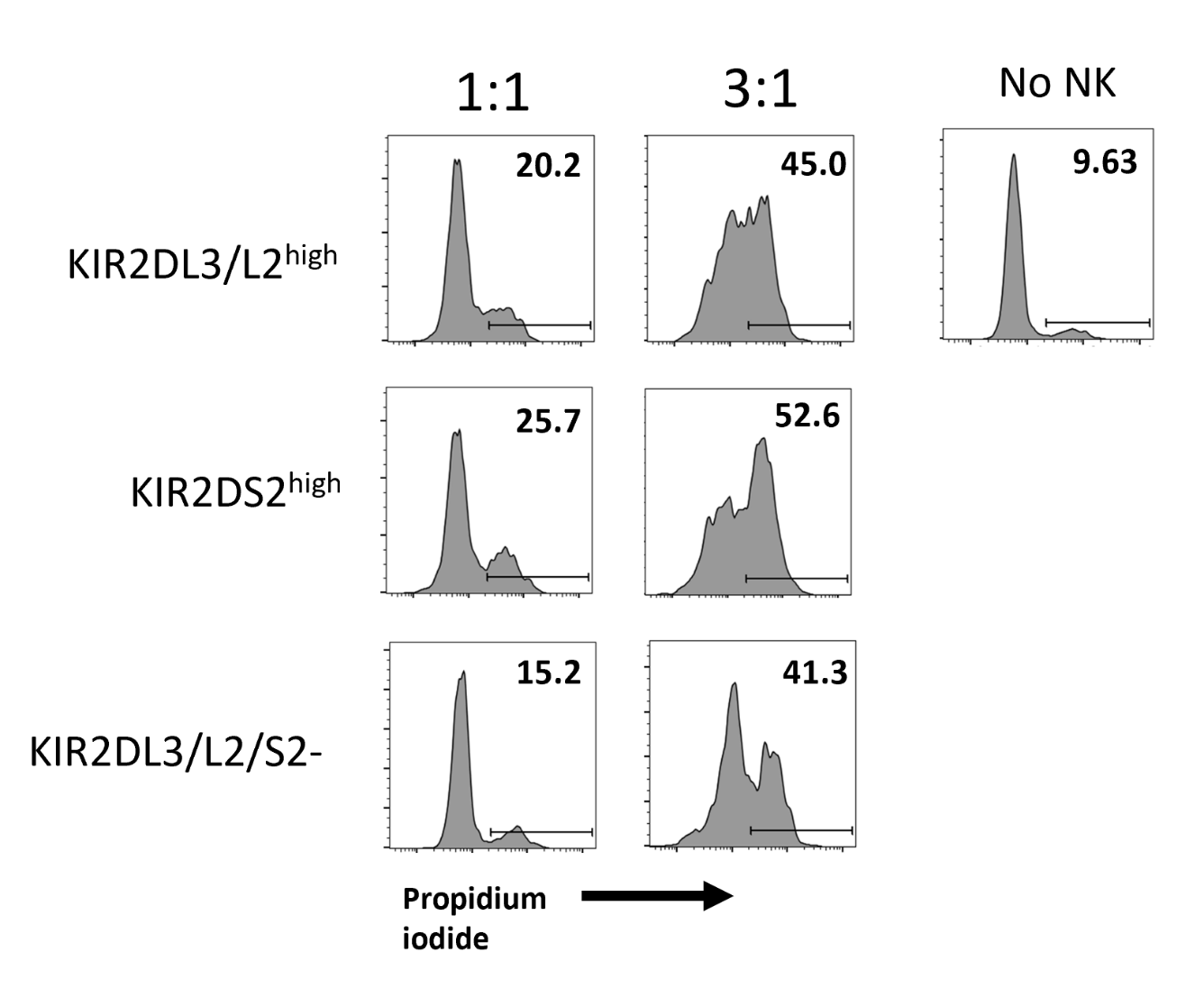 |
| --- |
| **Supplementary Figure 3: KIR2DS2^high^ NK cells induce greater lysis of target cells compared to KIR2DL3/L2^high^ and KIR2DL3/L2/S2- NK cells.**  Healthy donor NK cells were sorted into KIR2DS2^high^, KIR2DL3/L2^high^ and KIR2DL3/L2/S2- subpopulations by FACS before co-culture with CellTrace Violet-labelled 721.221 target cells at a 1:1 or 3:1 effector:target (E:T) ratio for 4 hours. The proportion of target cells lysed by the NK cells was measured by propidium iodide. Representative of 3 donors. |

|  |
| --- |
| **Supplementary Figure 4: NK cells expand in the Miltenyi Biotech NK expansion medium with IL-2 *ex vivo*.**  Primary human NK cells (CD3-CD56+) were isolated from healthy donor bulk PBMC on day 0 using the Miltenyi Biotech human NK cell isolation kit with. NK cells were then expanded ex vivo using Miltenyi Biotech NK cell MACS medium with IL-2 (expansion media). Expansion media was added every 2-3 days. Total cell counts were monitored on days 7, 10, 12 and 14. |

| **A** | **B** | **C** | **D** |
| --- | --- | --- | --- |
|  |  |  |    |
| **Supplementary Figure 5: Expression of activating receptors on the surface of KIR2DS2^high^, KIR2DL3/L2^high^ and KIR2DL3/L2/S2- NK cells before and after expansion with IL-2.**  NK cells were isolated from healthy donor PBMCs and cultured in NK MACS medium with IL-2 (expansion media) for 14 days. Expansion media was added every 2-3 days. Expression of activating receptors (A) NKp30, (B) NKp46, (C) NKG2D and (D) CD57 was measured by flow cytometry on days 0 and 14 of expansion (n=3). Analysed by two-way ANOVA using Graphpad PRISM. P<0.001***, p<0.0001****. | | | |
